# (*N*-Phenyl­thio­urea-κ*S*)bis­(tri­phenylphosphane-κ*P*)silver(I) nitrate

**DOI:** 10.1107/S1600536814014147

**Published:** 2014-06-25

**Authors:** Sofia Mekarat, Chaveng Pakawatchai, Saowanit Saithong

**Affiliations:** a Faculty of Science and Technology, Princess of Naradhiwas University, Narathiwat, 96000, Thailand; b Department of Chemistry and Center of Excellence for Innovation in Chemistry, Faculty of Science, Prince of Songkla University, Hat Yai, Songkhla 90112, Thailand

**Keywords:** *N-*phenyl­thio­urea, tri­phenyl­phosphane, silver(I)nitrate, crystal structure

## Abstract

In the title salt, [Ag(C_7_H_8_N_2_S)(C_18_H_15_P)_2_]NO_3_, the coordination geometry about the Ag^I^ atom is shallow trigonal pyramidal, with the metal atom displaced by 0.372 (1) Å from the plane of the P and S atoms. In the crystal, the cations are linked to the anions by N—H⋯O hydrogen bonds, generating tetra­mers (two cations and two anions), which feature *R*_2_^2^(8) and *R*_4_^4^(8) loops. The cations are linked by weak C—H⋯π inter­actions, generating a three-dimensional network.

## Related literature   

For properties of mixed-ligand *d* ^10^ metal(I) complexes, see: Oshio *et al.* (1996 ▶); Zheng *et al.* (2001 ▶); Sewead *et al.* (2003 ▶); Isab *et al.* (2010 ▶). For structural studies of mixed-ligand complexes of tri­phenyl­phosphane and thione ligands, see: Skoulika *et al.* (1991 ▶); Aslanidis *et al.* (1997 ▶); Ghassemzadeh *et al.*(2004 ▶); Nimthong *et al.* (2008 ▶); Isab *et al.* (2010 ▶).
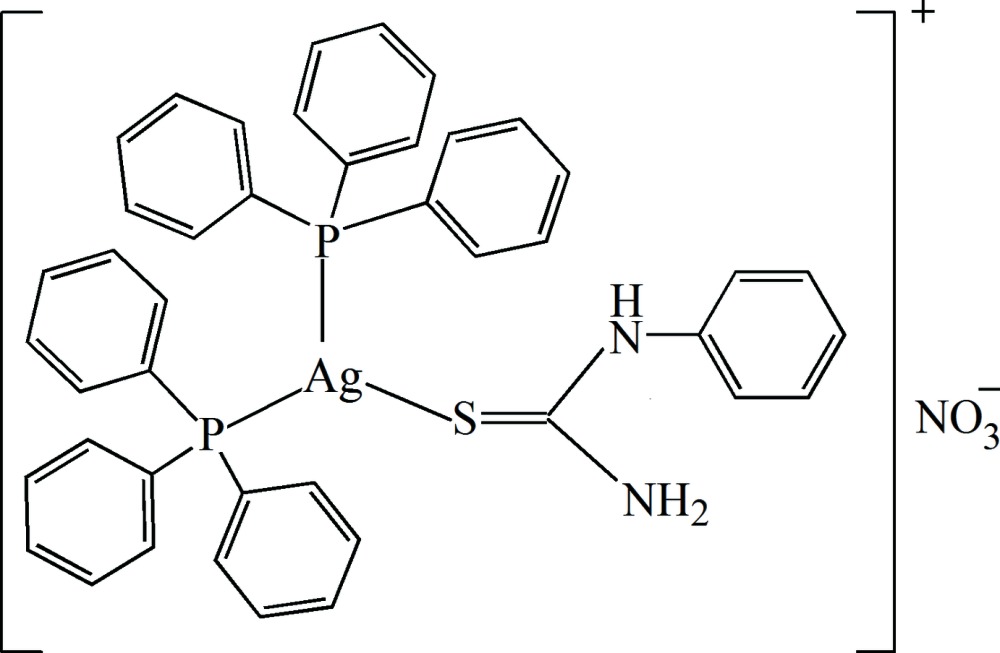


## Experimental   

### 

#### Crystal data  

[Ag(C_7_H_8_N_2_S)(C_18_H_15_P)_2_]NO_3_
*M* *_r_* = 846.63
Monoclinic, 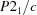

*a* = 13.6113 (5) Å
*b* = 10.6431 (4) Å
*c* = 26.4365 (10) Å
β = 96.068 (1)°
*V* = 3808.3 (2) Å^3^
*Z* = 4
Mo *K*α radiation
μ = 0.71 mm^−1^
*T* = 173 K
0.27 × 0.14 × 0.08 mm

#### Data collection  

Bruker SMART CCD diffractometer
Absorption correction: multi-scan (*SADABS*; Bruker, 2003 ▶) *T*_min_ = 0.863, *T*_max_ = 1.000
44417 measured reflections
9196 independent reflections
8261 reflections with *I* > 2σ(*I*)
*R*_int_ = 0.033

#### Refinement  

*R*[*F* ^2^ > 2σ(*F* ^2^)] = 0.028
*wR*(*F* ^2^) = 0.064
*S* = 1.05
9196 reflections
478 parameters
H-atom parameters constrained
Δρ_max_ = 0.55 e Å^−3^
Δρ_min_ = −0.26 e Å^−3^

### 

Data collection: *SMART* (Bruker, 2003 ▶); cell refinement: *SAINT* (Bruker, 2003 ▶); data reduction: *SAINT*; program(s) used to solve structure: *SHELXS2013* (Sheldrick, 2008 ▶); program(s) used to refine structure: *SHELXL2013* (Sheldrick, 2008 ▶); molecular graphics: *Mercury* (Macrae *et al.*, 2008 ▶); software used to prepare material for publication: *WinGX* (Farrugia, 2012 ▶) and *publCIF* (Westrip, 2010) ▶.

## Supplementary Material

Crystal structure: contains datablock(s) I, New_Global_Publ_Block. DOI: 10.1107/S1600536814014147/hb7242sup1.cif

Structure factors: contains datablock(s) I. DOI: 10.1107/S1600536814014147/hb7242Isup2.hkl

CCDC reference: 1008627

Additional supporting information:  crystallographic information; 3D view; checkCIF report

## Figures and Tables

**Table 1:** Hydrogen-bond geometry (Å, °) *Cg*2 is the centroid of the C11–C16 ring.

*D*—H⋯*A*	*D*—H	H⋯*A*	*D*⋯*A*	*D*—H⋯*A*
N1—H1*A*⋯O3^i^	0.86	2.02	2.877 (2)	180
N1—H1*B*⋯O3^ii^	0.86	2.17	2.921 (2)	145
N2—H2⋯O1^i^	0.86	1.97	2.823 (2)	171
C35—H35⋯*Cg*2^iii^	0.93	2.97	3.746 (2)	142
C54—H54⋯*Cg*2^iv^	0.93	2.82	3.531 (2)	134

Symmetry codes: (i) 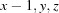; (ii) 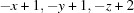; (iii) 
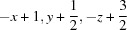
; (iv) 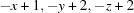.

**Fig. 1.**
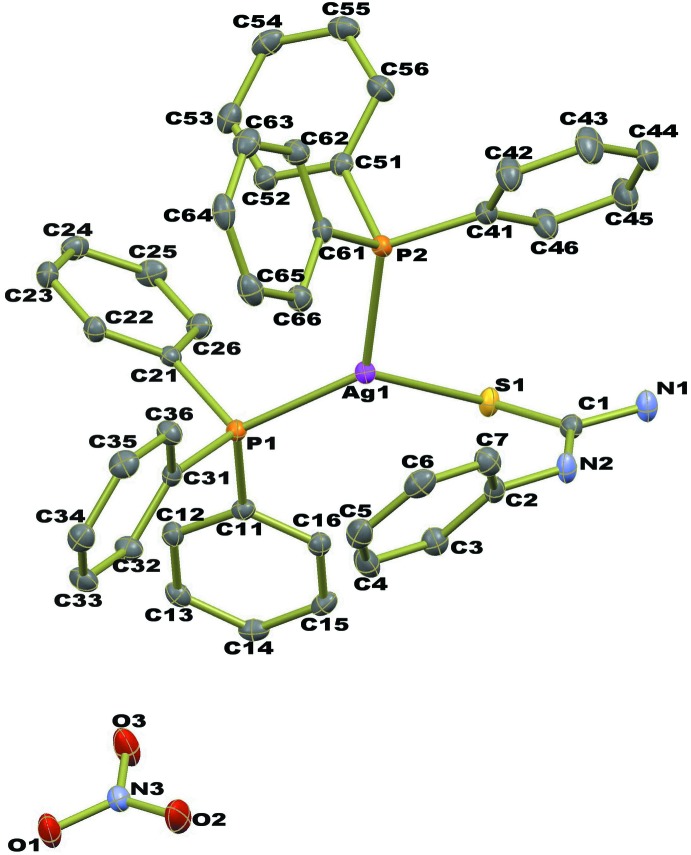
The molecular structure of [Ag(ptu)(PPh3)2]NO3 complex. Displacement ellipsoids are shown at 50% probability level.

**Fig. 2.**
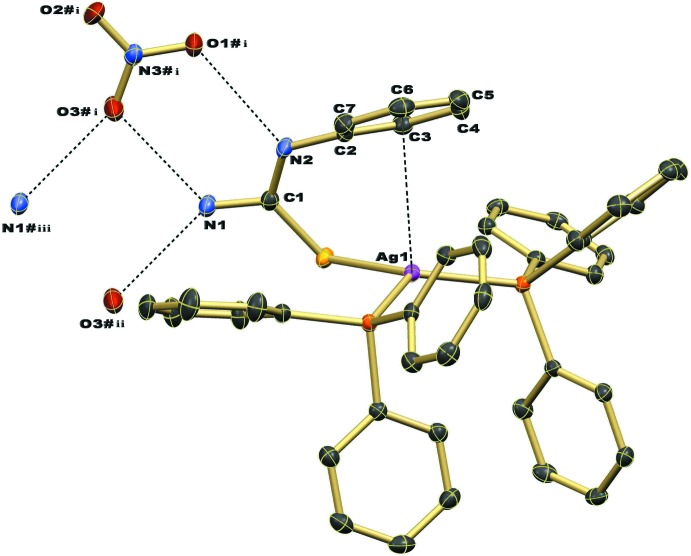
The hydrogen bonding interactions of [Ag(ptu)(PPh3)2]NO3 complex (#i: x - 1, y, z, #ii: 1 - x, 1 - y, 2 - z, #iii: -x, 1 - y,2 - z).

**Fig. 3.**
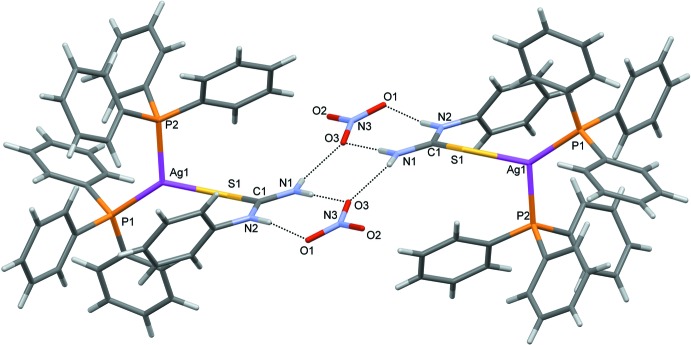
The cyclic of hydrogen bonding interactions containing two R22(8) and one R44(8).

**Fig. 4.**
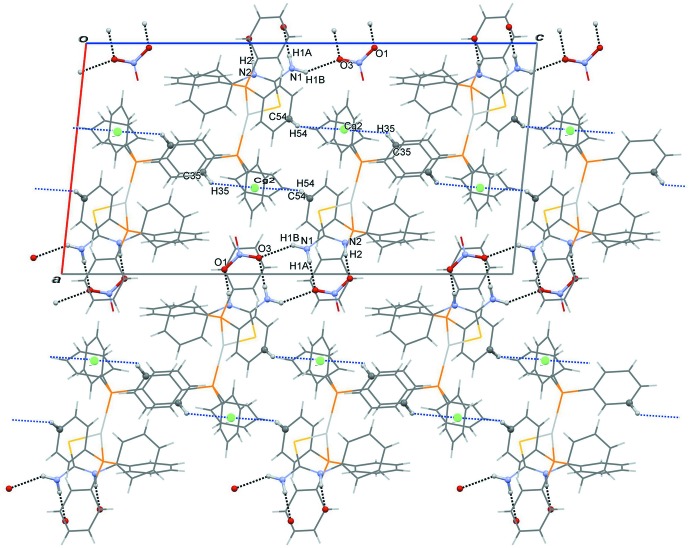
The three-dimensional supramolecular interactions in crystal packing.

## supplementary crystallographic information

### S1. Chemical context

Mixed-ligand complexes of group 11 metals dispaly many properties such as magnetism (Oshio *et al.*, 1996); microporousity (Zheng *et al.*, 2001); luminescence (Sewead *et al.*, 2003) and anti­microbial activities (Isab *et al.*,2010). In our earlier work, we synthesized and characterized the neutral monomeric copper(I) complex containing mixed ligands of tri­phenyl­phosphane (PPh_3_: C_18_H_15_P) and *N*-phynyl­thio­urea (ptu : C_7_H_8_N_2_S), [CuI(ptu)(PPh_3_)_2_] (Nimthong *et al.*, 2008). As part of our continuing studies in this area, we now describe the synthesis and structure of the title compound, [Ag(ptu)(PPh_3_)_2_]NO_3_ (Scheme I).

### S2. Structural commentary

Unlike the previous complex mentioned above (Nimthong *et al.*, 2008), this complex is an ionic complex and it crystallizes in monoclinic system space group *P*2_1_/*c*. The structure consists of the discrete mononuclear [Ag(ptu)(PPh_3_)_2_]^+^cation and the NO_3_^-^ anion which is similar to those [Ag(PPh_3_)_2_(pymtH)]NO_3_ (Aslanidis *et al.*, 1997). A perspective view of the molecular structure of [Ag(ptu)(PPh_3_)_2_]NO_3_ with atomic labeling is given in Figure 1. The cation part contains silver(I) atom trigonally coordinated by two phospho­rus atoms from two tri­phenyl­phosphane molecules and one sulfur atom from *N*–phenyl­thio­urea molecule similar to found in those silver oxyanion complexes containing mixed PPh_3_/heterocyclic thione ligands (Aslanidis *et al.*, 1997; Ghassemzadeh *et al.*, 2004). The Ag–P bond lengths of 2.4645 (5) and 2.4693 (4)Å are similar to the values of 2.455 (1) and 2.447 (1) Å observed in [Ag(PPh_3_)_2_(pymtH)]NO_3_ (Aslanidis *et al.*, 1997), however, these values are slightly different from the values of 2.458 (2) and 2.507 (2) Å compared to [Ag(TAMTTO)(PPh_3_)_2_]NO_3_.1.5THF (Ghassemzadeh *et al.*, 2004) because of the massive and steric effect of TAMTTO heterocyclic ligand. The Ag–S bond length [2.5307 (7) Å] is shorter than in those complexes [Ag(PPh_3_)_2_(pymtH)]NO_3_ [2.573 (1)Å] and [Ag(TAMTTO)(PPh_3_)_2_]NO_3_.1.5 THF [2.592 (2) Å] (Aslanidis *et al.*, 1997; Ghassemzadeh *et al.*, 2004 ). The P(1)–Ag–P(2), P(1)–Ag–S(1) and P(2)–Ag–S(1) bond angles are 127.55 (1)° ,113.02 (1)° and 112.69 (1)°, respectively. Due to the steric crowding of six phenyl rings from two bulky tri­phenyl phosphane ligands and the *π*(CH)···Ag inter­action [3.314 Å] between the centriod of phenyl ring (C2—C7) of the *N*–phenyl­thio­urea and metal atom, the silver centre atom deviates from idealized trigonal planar with this atom lying *ca* 0.372 (1) Å out of the P_2_S plane. For the anion, although the oxygen atoms of the nitrate have no influence on coordination, they have great influence on the crystal packing of the complex. It is nearly planar with the bond angles around the nitro­gen atom ranging from 119.01 (1)-120.53 (1)° and N(3)–O bond distances are 1.231 (2) – 1.264 (2) Å.

### S3. Supra­molecular features

For the crystal packing, each [Ag(ptu)(PPh_3_)_2_]^+^ cation is connected to another adjacent cationic part via hydrogen bonding inter­actions, N–H···O, which are observed between amino and amide groups and nitrate oxygen atoms generate a cyclic hydrogen bond inter­actions, two *R_2_^2^(8*) graph sets for cationic-anionic inter­actions and one *R_4_^4^(8)* graph set for anionic-anionic inter­action, [ N(1)–H(1A)···O(3)^i^ : 2.877 (2)Å, N(1)–H(1B)···O(3)^ii^ : 2.921 (2)Å, N(2)–H(2)···O(1) : 2.823 (2) Å and symmetry code : (i) x-1,y,z, (ii) -x+1,-y+1,-z+2] as depicted in Figure 2 and 3. In addition, the cationic parts are linked together by the CH···π inter­actions between the phenyl rings with the distance of 3.746 (2) Å for C35–H35···Cg2 and 3.531 (2) Å for C54–H54···Cg2 [Cg2 : C11–C16] generating the three dimensional supra­molecular network. All inter­actions are depicted in Figure 4.

### S4. Synthesis and crystallization

The mixture silver(I) nitrate and tri­phenyl­phosphane in ethanol was refluxed at the temperature *ca* 60-70 ^°^C for 2 h. After that, *N*-phenyl­thio­urea ligand was added to the clear mixture solution and then continued to reflux futher for 3 h. The clear filtration was kept and left to evaporate slowly at ambient temperature. After several days, colorless blocks were obtained. The melting point of the complex is 192-194 ^°^C . Elemental analysis,calculated for [Ag(PPh_3_)_2_(ptu)]NO_3_ : C, 60.99;H, 4.52; N, 4.96; S, 3.78%, found: C, 65.16; H, 4.96; N, 5.16; S, 4.04%.

### S5. Refinement

Crystal data, data collection and structure refinement details are summarized in Table 1. The structures were solved by direct methods and refined by a full-matrix least-squares procedure based on *F*^2^. All hydrogen atoms were placed in geometrically idealised positions and refined isotropically with a riding model for both of amine N [N—H = 0.86 Å and with *U_i_*_so_(H) =1.2*U*_eq_(N)] and phenyl ring C-*sp*^2^[C—H = 0.93 Å and with *U_i_*_so_(H) = 1.2*U*_eq_(C)]. ;

### Crystal data

**Table:**

[Ag(C_7_H_8_N_2_S)(C_18_H_15_P)_2_]NO_3_	*F*(000) = 1736
*M**_r_* = 846.63	*D*_x_ = 1.477 Mg m^−^^3^
Monoclinic, *P*2_1_/*c*	Mo *K*α radiation, λ = 0.71073 Å
*a* = 13.6113 (5) Å	Cell parameters from 13930 reflections
*b* = 10.6431 (4) Å	θ = 2.3–28.0°
*c* = 26.4365 (10) Å	µ = 0.71 mm^−^^1^
β = 96.068 (1)°	*T* = 173 K
*V* = 3808.3 (2) Å^3^	Block, colourless
*Z* = 4	0.27 × 0.14 × 0.08 mm

### Data collection

**Table:**

Bruker SMART CCD diffractometer	8261 reflections with *I* > 2σ(*I*)
Radiation source: fine-focus sealed tube	*R*_int_ = 0.033
Frames each covering 0.3 ° in ω scans	θ_max_ = 28.0°, θ_min_ = 1.5°
Absorption correction: multi-scan (*SADABS*; Bruker, 2003)	*h* = −17→17
*T*_min_ = 0.863, *T*_max_ = 1.000	*k* = −14→14
44417 measured reflections	*l* = −34→34
9196 independent reflections	

### Refinement

**Table:**

Refinement on *F*^2^	Primary atom site location: structure-invariant direct methods
Least-squares matrix: full	Secondary atom site location: difference Fourier map
*R*[*F*^2^ > 2σ(*F*^2^)] = 0.028	Hydrogen site location: inferred from neighbouring sites
*wR*(*F*^2^) = 0.064	H-atom parameters constrained
*S* = 1.05	*w* = 1/[σ^2^(*F*_o_^2^) + (0.0264*P*)^2^ + 2.6456*P*] where *P* = (*F*_o_^2^ + 2*F*_c_^2^)/3
9196 reflections	(Δ/σ)_max_ = 0.003
478 parameters	Δρ_max_ = 0.55 e Å^−^^3^
0 restraints	Δρ_min_ = −0.26 e Å^−^^3^

### Special details

**Table:**

Geometry. All e.s.d.'s (except the e.s.d. in the dihedral angle between two l.s. planes) are estimated using the full covariance matrix. The cell e.s.d.'s are taken into account individually in the estimation of e.s.d.'s in distances, angles and torsion angles; correlations between e.s.d.'s in cell parameters are only used when they are defined by crystal symmetry. An approximate (isotropic) treatment of cell e.s.d.'s is used for estimating e.s.d.'s involving l.s. planes.

### Fractional atomic coordinates and isotropic or equivalent isotropic displacement parameters (Å^2^)

**Table:**

	*x*	*y*	*z*	*U*_iso_*/*U*_eq_	
Ag1	0.30947 (2)	0.80538 (2)	0.87530 (2)	0.01346 (4)	
S1	0.27373 (3)	0.65461 (4)	0.94508 (2)	0.01679 (9)	
P1	0.48557 (3)	0.81150 (4)	0.86130 (2)	0.01253 (8)	
P2	0.18820 (3)	0.97824 (4)	0.86365 (2)	0.01356 (9)	
N1	0.10876 (11)	0.54151 (15)	0.96207 (6)	0.0205 (3)	
H1A	0.0553	0.5001	0.9535	0.025*	
H1B	0.1249	0.5609	0.9934	0.025*	
N2	0.13553 (11)	0.54123 (15)	0.87904 (6)	0.0195 (3)	
H2	0.0848	0.4926	0.8754	0.023*	
N3	0.91546 (11)	0.35396 (14)	0.88996 (6)	0.0200 (3)	
O1	0.98030 (10)	0.36618 (14)	0.85981 (5)	0.0285 (3)	
O2	0.83834 (11)	0.29694 (14)	0.87678 (6)	0.0339 (4)	
O3	0.92984 (10)	0.40319 (15)	0.93362 (5)	0.0320 (3)	
C1	0.16649 (13)	0.57551 (16)	0.92682 (6)	0.0166 (3)	
C2	0.17639 (13)	0.57559 (16)	0.83349 (6)	0.0165 (3)	
C3	0.27222 (13)	0.54423 (18)	0.82506 (7)	0.0203 (4)	
H3	0.3129	0.5015	0.8498	0.024*	
C4	0.30689 (14)	0.57740 (19)	0.77919 (7)	0.0246 (4)	
H4	0.3714	0.5580	0.7735	0.030*	
C5	0.24611 (15)	0.63894 (18)	0.74210 (7)	0.0244 (4)	
H5	0.2700	0.6612	0.7117	0.029*	
C6	0.14981 (15)	0.66765 (17)	0.75003 (7)	0.0233 (4)	
H6	0.1086	0.7080	0.7248	0.028*	
C7	0.11484 (14)	0.63599 (18)	0.79586 (7)	0.0212 (4)	
H7	0.0502	0.6553	0.8013	0.025*	
C11	0.56534 (12)	0.68448 (15)	0.88711 (6)	0.0142 (3)	
C12	0.66839 (13)	0.69391 (16)	0.88805 (6)	0.0160 (3)	
H12	0.6962	0.7659	0.8756	0.019*	
C13	0.72891 (13)	0.59703 (17)	0.90730 (6)	0.0168 (3)	
H13	0.7970	0.6032	0.9070	0.020*	
C14	0.68800 (13)	0.49043 (16)	0.92701 (6)	0.0178 (3)	
H14	0.7285	0.4246	0.9394	0.021*	
C15	0.58661 (13)	0.48238 (16)	0.92812 (6)	0.0178 (3)	
H15	0.5596	0.4123	0.9425	0.021*	
C16	0.52496 (13)	0.57825 (16)	0.90794 (6)	0.0154 (3)	
H16	0.4569	0.5716	0.9083	0.018*	
C21	0.55372 (12)	0.94570 (15)	0.89023 (6)	0.0136 (3)	
C22	0.59006 (12)	1.04369 (16)	0.86294 (6)	0.0161 (3)	
H22	0.5778	1.0452	0.8277	0.019*	
C23	0.64480 (13)	1.13972 (17)	0.88827 (7)	0.0182 (3)	
H23	0.6684	1.2056	0.8698	0.022*	
C24	0.66425 (13)	1.13752 (17)	0.94081 (7)	0.0195 (4)	
H24	0.7028	1.2001	0.9575	0.023*	
C25	0.62590 (14)	1.04144 (17)	0.96837 (7)	0.0211 (4)	
H25	0.6374	1.0408	1.0037	0.025*	
C26	0.57049 (13)	0.94653 (17)	0.94329 (6)	0.0187 (4)	
H26	0.5443	0.8829	0.9619	0.022*	
C31	0.50225 (12)	0.81928 (15)	0.79374 (6)	0.0135 (3)	
C32	0.57450 (13)	0.75263 (17)	0.77151 (7)	0.0183 (3)	
H32	0.6192	0.7031	0.7917	0.022*	
C33	0.58030 (14)	0.75957 (18)	0.71953 (7)	0.0213 (4)	
H33	0.6288	0.7147	0.7050	0.026*	
C34	0.51386 (14)	0.83325 (17)	0.68905 (7)	0.0211 (4)	
H34	0.5178	0.8377	0.6542	0.025*	
C35	0.44182 (14)	0.90008 (17)	0.71074 (7)	0.0208 (4)	
H35	0.3974	0.9498	0.6904	0.025*	
C36	0.43562 (13)	0.89312 (17)	0.76278 (7)	0.0181 (3)	
H36	0.3868	0.9379	0.7771	0.022*	
C41	0.06641 (13)	0.94975 (16)	0.88407 (7)	0.0166 (3)	
C42	−0.01987 (14)	0.99999 (19)	0.85980 (8)	0.0263 (4)	
H42	−0.0179	1.0465	0.8301	0.032*	
C43	−0.10945 (14)	0.9812 (2)	0.87964 (9)	0.0314 (5)	
H43	−0.1670	1.0157	0.8633	0.038*	
C44	−0.11355 (14)	0.91173 (19)	0.92340 (8)	0.0270 (4)	
H44	−0.1735	0.8999	0.9367	0.032*	
C45	−0.02773 (15)	0.8597 (2)	0.94747 (8)	0.0288 (4)	
H45	−0.0301	0.8124	0.9769	0.035*	
C46	0.06196 (14)	0.87786 (19)	0.92784 (7)	0.0242 (4)	
H46	0.1192	0.8419	0.9440	0.029*	
C51	0.23925 (12)	1.10092 (15)	0.90686 (6)	0.0149 (3)	
C52	0.33905 (13)	1.12973 (16)	0.90577 (7)	0.0178 (3)	
H52	0.3765	1.0845	0.8846	0.021*	
C53	0.38268 (14)	1.22486 (17)	0.93583 (7)	0.0216 (4)	
H53	0.4492	1.2435	0.9347	0.026*	
C54	0.32787 (15)	1.29242 (17)	0.96757 (7)	0.0237 (4)	
H54	0.3572	1.3568	0.9876	0.028*	
C55	0.22880 (15)	1.26367 (18)	0.96936 (7)	0.0237 (4)	
H55	0.1920	1.3084	0.9910	0.028*	
C56	0.18420 (14)	1.16877 (17)	0.93912 (7)	0.0206 (4)	
H56	0.1177	1.1504	0.9404	0.025*	
C61	0.16834 (12)	1.06410 (16)	0.80372 (6)	0.0153 (3)	
C62	0.14519 (14)	1.19195 (17)	0.80202 (7)	0.0200 (4)	
H62	0.1353	1.2345	0.8318	0.024*	
C63	0.13683 (14)	1.25589 (18)	0.75580 (7)	0.0233 (4)	
H63	0.1217	1.3411	0.7548	0.028*	
C64	0.15107 (14)	1.19286 (19)	0.71128 (7)	0.0230 (4)	
H64	0.1459	1.2358	0.6805	0.028*	
C65	0.17304 (14)	1.06573 (19)	0.71274 (7)	0.0237 (4)	
H65	0.1819	1.0231	0.6828	0.028*	
C66	0.18175 (13)	1.00199 (18)	0.75869 (7)	0.0192 (4)	
H66	0.1967	0.9167	0.7594	0.023*	

### Atomic displacement parameters (Å^2^)

**Table:**

	*U*^11^	*U*^22^	*U*^33^	*U*^12^	*U*^13^	*U*^23^
Ag1	0.01252 (7)	0.01365 (6)	0.01457 (6)	−0.00079 (4)	0.00304 (4)	0.00008 (5)
S1	0.0166 (2)	0.0204 (2)	0.01331 (19)	−0.00489 (16)	0.00144 (15)	0.00208 (16)
P1	0.01206 (19)	0.01232 (19)	0.01342 (19)	−0.00013 (15)	0.00226 (15)	0.00091 (15)
P2	0.0125 (2)	0.0139 (2)	0.0147 (2)	0.00012 (15)	0.00337 (16)	0.00002 (16)
N1	0.0186 (7)	0.0264 (8)	0.0170 (7)	−0.0071 (6)	0.0043 (6)	0.0003 (6)
N2	0.0160 (7)	0.0250 (8)	0.0178 (7)	−0.0085 (6)	0.0036 (6)	−0.0010 (6)
N3	0.0158 (7)	0.0192 (7)	0.0258 (8)	−0.0008 (6)	0.0057 (6)	−0.0009 (6)
O1	0.0226 (7)	0.0375 (8)	0.0274 (7)	−0.0110 (6)	0.0122 (6)	−0.0076 (6)
O2	0.0222 (7)	0.0362 (8)	0.0449 (9)	−0.0150 (6)	0.0110 (6)	−0.0154 (7)
O3	0.0241 (7)	0.0499 (9)	0.0235 (7)	−0.0120 (7)	0.0093 (6)	−0.0110 (7)
C1	0.0168 (8)	0.0157 (8)	0.0175 (8)	−0.0003 (6)	0.0033 (6)	0.0020 (6)
C2	0.0171 (8)	0.0184 (8)	0.0142 (8)	−0.0059 (7)	0.0033 (6)	−0.0021 (6)
C3	0.0192 (9)	0.0233 (9)	0.0179 (8)	−0.0016 (7)	0.0004 (7)	−0.0015 (7)
C4	0.0212 (9)	0.0318 (10)	0.0221 (9)	−0.0022 (8)	0.0086 (7)	−0.0056 (8)
C5	0.0331 (11)	0.0249 (10)	0.0161 (9)	−0.0078 (8)	0.0076 (8)	−0.0037 (7)
C6	0.0300 (10)	0.0204 (9)	0.0183 (9)	−0.0027 (8)	−0.0032 (7)	−0.0008 (7)
C7	0.0168 (9)	0.0253 (9)	0.0214 (9)	−0.0018 (7)	0.0007 (7)	−0.0029 (7)
C11	0.0164 (8)	0.0131 (7)	0.0129 (7)	0.0005 (6)	0.0013 (6)	−0.0007 (6)
C12	0.0174 (8)	0.0162 (8)	0.0150 (8)	−0.0011 (6)	0.0037 (6)	0.0011 (6)
C13	0.0144 (8)	0.0223 (9)	0.0134 (8)	0.0021 (7)	0.0010 (6)	−0.0021 (7)
C14	0.0231 (9)	0.0159 (8)	0.0136 (8)	0.0049 (7)	−0.0012 (7)	−0.0013 (6)
C15	0.0244 (9)	0.0123 (8)	0.0164 (8)	−0.0021 (7)	0.0010 (7)	0.0011 (6)
C16	0.0159 (8)	0.0159 (8)	0.0144 (8)	−0.0018 (6)	0.0020 (6)	−0.0010 (6)
C21	0.0114 (7)	0.0135 (8)	0.0161 (8)	0.0015 (6)	0.0022 (6)	−0.0014 (6)
C22	0.0169 (8)	0.0187 (8)	0.0128 (8)	−0.0017 (7)	0.0027 (6)	−0.0001 (6)
C23	0.0183 (8)	0.0175 (8)	0.0194 (8)	−0.0029 (7)	0.0051 (7)	0.0014 (7)
C24	0.0186 (9)	0.0173 (8)	0.0220 (9)	−0.0014 (7)	−0.0009 (7)	−0.0050 (7)
C25	0.0288 (10)	0.0213 (9)	0.0127 (8)	0.0016 (7)	−0.0007 (7)	−0.0005 (7)
C26	0.0244 (9)	0.0168 (8)	0.0155 (8)	0.0005 (7)	0.0043 (7)	0.0027 (7)
C31	0.0139 (8)	0.0137 (8)	0.0127 (7)	−0.0033 (6)	0.0012 (6)	−0.0001 (6)
C32	0.0187 (9)	0.0188 (8)	0.0172 (8)	0.0037 (7)	0.0010 (7)	0.0002 (7)
C33	0.0233 (9)	0.0237 (9)	0.0177 (8)	0.0033 (7)	0.0064 (7)	−0.0028 (7)
C34	0.0273 (10)	0.0220 (9)	0.0139 (8)	−0.0034 (7)	0.0022 (7)	−0.0007 (7)
C35	0.0234 (9)	0.0203 (9)	0.0177 (8)	0.0018 (7)	−0.0023 (7)	0.0026 (7)
C36	0.0163 (8)	0.0194 (8)	0.0187 (8)	0.0023 (7)	0.0024 (7)	0.0001 (7)
C41	0.0157 (8)	0.0154 (8)	0.0195 (8)	−0.0010 (6)	0.0052 (7)	−0.0014 (7)
C42	0.0187 (9)	0.0299 (10)	0.0304 (10)	0.0013 (8)	0.0036 (8)	0.0087 (8)
C43	0.0140 (9)	0.0384 (12)	0.0419 (12)	0.0027 (8)	0.0038 (8)	0.0075 (10)
C44	0.0184 (9)	0.0279 (10)	0.0370 (11)	−0.0036 (8)	0.0127 (8)	−0.0025 (9)
C45	0.0280 (10)	0.0315 (11)	0.0292 (10)	0.0013 (8)	0.0134 (8)	0.0067 (9)
C46	0.0187 (9)	0.0277 (10)	0.0273 (10)	0.0044 (7)	0.0074 (7)	0.0059 (8)
C51	0.0166 (8)	0.0140 (8)	0.0140 (8)	0.0008 (6)	0.0014 (6)	0.0021 (6)
C52	0.0204 (9)	0.0167 (8)	0.0169 (8)	0.0002 (7)	0.0042 (7)	0.0001 (7)
C53	0.0221 (9)	0.0205 (9)	0.0219 (9)	−0.0063 (7)	0.0012 (7)	0.0018 (7)
C54	0.0344 (11)	0.0175 (9)	0.0181 (9)	−0.0032 (8)	−0.0020 (8)	−0.0022 (7)
C55	0.0294 (10)	0.0224 (9)	0.0199 (9)	0.0038 (8)	0.0055 (8)	−0.0043 (7)
C56	0.0202 (9)	0.0229 (9)	0.0192 (9)	0.0020 (7)	0.0042 (7)	−0.0015 (7)
C61	0.0117 (8)	0.0179 (8)	0.0165 (8)	−0.0014 (6)	0.0027 (6)	0.0017 (6)
C62	0.0212 (9)	0.0206 (9)	0.0184 (8)	0.0013 (7)	0.0033 (7)	0.0000 (7)
C63	0.0243 (10)	0.0200 (9)	0.0253 (9)	−0.0001 (7)	0.0007 (8)	0.0057 (8)
C64	0.0176 (9)	0.0336 (10)	0.0180 (9)	−0.0039 (8)	0.0021 (7)	0.0077 (8)
C65	0.0208 (9)	0.0346 (11)	0.0162 (8)	−0.0033 (8)	0.0041 (7)	−0.0027 (8)
C66	0.0174 (8)	0.0211 (9)	0.0194 (8)	−0.0014 (7)	0.0028 (7)	−0.0024 (7)

### Geometric parameters (Å, º)

**Table:**

Ag1—P1	2.4645 (5)	C24—H24	0.9300
Ag1—P2	2.4693 (4)	C25—C26	1.387 (3)
Ag1—S1	2.5307 (4)	C25—H25	0.9300
S1—C1	1.7098 (18)	C26—H26	0.9300
P1—C11	1.8208 (17)	C31—C32	1.392 (2)
P1—C31	1.8260 (17)	C31—C36	1.398 (2)
P1—C21	1.8262 (17)	C32—C33	1.387 (2)
P2—C41	1.8222 (18)	C32—H32	0.9300
P2—C51	1.8235 (17)	C33—C34	1.389 (3)
P2—C61	1.8241 (17)	C33—H33	0.9300
N1—C1	1.331 (2)	C34—C35	1.384 (3)
N1—H1A	0.8600	C34—H34	0.9300
N1—H1B	0.8600	C35—C36	1.389 (2)
N2—C1	1.339 (2)	C35—H35	0.9300
N2—C2	1.426 (2)	C36—H36	0.9300
N2—H2	0.8600	C41—C42	1.385 (3)
N3—O2	1.231 (2)	C41—C46	1.394 (3)
N3—O1	1.2568 (19)	C42—C43	1.392 (3)
N3—O3	1.264 (2)	C42—H42	0.9300
C2—C3	1.387 (2)	C43—C44	1.379 (3)
C2—C7	1.389 (2)	C43—H43	0.9300
C3—C4	1.392 (3)	C44—C45	1.385 (3)
C3—H3	0.9300	C44—H44	0.9300
C4—C5	1.380 (3)	C45—C46	1.390 (3)
C4—H4	0.9300	C45—H45	0.9300
C5—C6	1.383 (3)	C46—H46	0.9300
C5—H5	0.9300	C51—C56	1.395 (2)
C6—C7	1.389 (3)	C51—C52	1.396 (2)
C6—H6	0.9300	C52—C53	1.382 (2)
C7—H7	0.9300	C52—H52	0.9300
C11—C16	1.396 (2)	C53—C54	1.382 (3)
C11—C12	1.404 (2)	C53—H53	0.9300
C12—C13	1.383 (2)	C54—C55	1.388 (3)
C12—H12	0.9300	C54—H54	0.9300
C13—C14	1.389 (2)	C55—C56	1.387 (3)
C13—H13	0.9300	C55—H55	0.9300
C14—C15	1.386 (3)	C56—H56	0.9300
C14—H14	0.9300	C61—C66	1.391 (2)
C15—C16	1.391 (2)	C61—C62	1.396 (2)
C15—H15	0.9300	C62—C63	1.393 (3)
C16—H16	0.9300	C62—H62	0.9300
C21—C22	1.389 (2)	C63—C64	1.386 (3)
C21—C26	1.397 (2)	C63—H63	0.9300
C22—C23	1.394 (2)	C64—C65	1.385 (3)
C22—H22	0.9300	C64—H64	0.9300
C23—C24	1.387 (2)	C65—C66	1.386 (3)
C23—H23	0.9300	C65—H65	0.9300
C24—C25	1.389 (3)	C66—H66	0.9300
			
P1—Ag1—P2	127.556 (15)	C26—C25—H25	120.0
P1—Ag1—S1	113.029 (15)	C24—C25—H25	120.0
P2—Ag1—S1	112.694 (15)	C25—C26—C21	120.50 (16)
C1—S1—Ag1	109.30 (6)	C25—C26—H26	119.7
C11—P1—C31	105.62 (8)	C21—C26—H26	119.7
C11—P1—C21	99.64 (8)	C32—C31—C36	118.88 (15)
C31—P1—C21	105.30 (8)	C32—C31—P1	123.80 (13)
C11—P1—Ag1	118.36 (6)	C36—C31—P1	117.27 (13)
C31—P1—Ag1	111.81 (6)	C33—C32—C31	120.52 (16)
C21—P1—Ag1	114.63 (5)	C33—C32—H32	119.7
C41—P2—C51	103.41 (8)	C31—C32—H32	119.7
C41—P2—C61	106.55 (8)	C32—C33—C34	120.23 (17)
C51—P2—C61	101.36 (8)	C32—C33—H33	119.9
C41—P2—Ag1	117.14 (6)	C34—C33—H33	119.9
C51—P2—Ag1	104.48 (6)	C35—C34—C33	119.75 (17)
C61—P2—Ag1	121.15 (6)	C35—C34—H34	120.1
C1—N1—H1A	120.0	C33—C34—H34	120.1
C1—N1—H1B	120.0	C34—C35—C36	120.19 (17)
H1A—N1—H1B	120.0	C34—C35—H35	119.9
C1—N2—C2	127.91 (15)	C36—C35—H35	119.9
C1—N2—H2	116.0	C35—C36—C31	120.42 (16)
C2—N2—H2	116.0	C35—C36—H36	119.8
O2—N3—O1	120.45 (16)	C31—C36—H36	119.8
O2—N3—O3	120.53 (15)	C42—C41—C46	119.18 (17)
O1—N3—O3	119.01 (15)	C42—C41—P2	123.50 (14)
N1—C1—N2	115.86 (16)	C46—C41—P2	117.26 (13)
N1—C1—S1	119.06 (13)	C41—C42—C43	120.24 (18)
N2—C1—S1	125.06 (13)	C41—C42—H42	119.9
C3—C2—C7	120.16 (16)	C43—C42—H42	119.9
C3—C2—N2	122.09 (16)	C44—C43—C42	120.54 (19)
C7—C2—N2	117.64 (16)	C44—C43—H43	119.7
C2—C3—C4	119.35 (17)	C42—C43—H43	119.7
C2—C3—H3	120.3	C43—C44—C45	119.52 (18)
C4—C3—H3	120.3	C43—C44—H44	120.2
C5—C4—C3	120.43 (18)	C45—C44—H44	120.2
C5—C4—H4	119.8	C44—C45—C46	120.28 (19)
C3—C4—H4	119.8	C44—C45—H45	119.9
C4—C5—C6	120.25 (18)	C46—C45—H45	119.9
C4—C5—H5	119.9	C45—C46—C41	120.23 (18)
C6—C5—H5	119.9	C45—C46—H46	119.9
C5—C6—C7	119.74 (18)	C41—C46—H46	119.9
C5—C6—H6	120.1	C56—C51—C52	119.07 (16)
C7—C6—H6	120.1	C56—C51—P2	124.06 (13)
C2—C7—C6	120.05 (17)	C52—C51—P2	116.84 (13)
C2—C7—H7	120.0	C53—C52—C51	120.60 (17)
C6—C7—H7	120.0	C53—C52—H52	119.7
C16—C11—C12	119.03 (15)	C51—C52—H52	119.7
C16—C11—P1	120.35 (13)	C52—C53—C54	120.24 (18)
C12—C11—P1	120.58 (13)	C52—C53—H53	119.9
C13—C12—C11	120.56 (16)	C54—C53—H53	119.9
C13—C12—H12	119.7	C53—C54—C55	119.64 (17)
C11—C12—H12	119.7	C53—C54—H54	120.2
C12—C13—C14	120.01 (16)	C55—C54—H54	120.2
C12—C13—H13	120.0	C56—C55—C54	120.54 (18)
C14—C13—H13	120.0	C56—C55—H55	119.7
C15—C14—C13	119.86 (16)	C54—C55—H55	119.7
C15—C14—H14	120.1	C55—C56—C51	119.91 (17)
C13—C14—H14	120.1	C55—C56—H56	120.0
C14—C15—C16	120.54 (16)	C51—C56—H56	120.0
C14—C15—H15	119.7	C66—C61—C62	119.03 (16)
C16—C15—H15	119.7	C66—C61—P2	118.99 (13)
C15—C16—C11	119.92 (16)	C62—C61—P2	121.89 (13)
C15—C16—H16	120.0	C63—C62—C61	120.10 (17)
C11—C16—H16	120.0	C63—C62—H62	119.9
C22—C21—C26	119.17 (15)	C61—C62—H62	119.9
C22—C21—P1	124.26 (13)	C64—C63—C62	120.18 (18)
C26—C21—P1	116.56 (13)	C64—C63—H63	119.9
C21—C22—C23	120.20 (16)	C62—C63—H63	119.9
C21—C22—H22	119.9	C65—C64—C63	119.90 (17)
C23—C22—H22	119.9	C65—C64—H64	120.1
C24—C23—C22	120.30 (16)	C63—C64—H64	120.1
C24—C23—H23	119.9	C64—C65—C66	120.05 (18)
C22—C23—H23	119.8	C64—C65—H65	120.0
C23—C24—C25	119.70 (16)	C66—C65—H65	120.0
C23—C24—H24	120.2	C65—C66—C61	120.74 (17)
C25—C24—H24	120.2	C65—C66—H66	119.6
C26—C25—C24	120.06 (16)	C61—C66—H66	119.6
			
C2—N2—C1—N1	172.88 (17)	P1—C31—C32—C33	177.53 (14)
C2—N2—C1—S1	−8.7 (3)	C31—C32—C33—C34	0.0 (3)
Ag1—S1—C1—N1	−147.45 (13)	C32—C33—C34—C35	0.1 (3)
Ag1—S1—C1—N2	34.16 (17)	C33—C34—C35—C36	−0.3 (3)
C1—N2—C2—C3	61.7 (3)	C34—C35—C36—C31	0.4 (3)
C1—N2—C2—C7	−122.0 (2)	C32—C31—C36—C35	−0.3 (3)
C7—C2—C3—C4	2.0 (3)	P1—C31—C36—C35	−177.86 (14)
N2—C2—C3—C4	178.19 (17)	C51—P2—C41—C42	99.52 (17)
C2—C3—C4—C5	−1.1 (3)	C61—P2—C41—C42	−6.87 (18)
C3—C4—C5—C6	−0.4 (3)	Ag1—P2—C41—C42	−146.22 (15)
C4—C5—C6—C7	1.0 (3)	C51—P2—C41—C46	−77.57 (15)
C3—C2—C7—C6	−1.4 (3)	C61—P2—C41—C46	176.04 (14)
N2—C2—C7—C6	−177.76 (16)	Ag1—P2—C41—C46	36.69 (16)
C5—C6—C7—C2	−0.1 (3)	C46—C41—C42—C43	1.5 (3)
C31—P1—C11—C16	−117.34 (14)	P2—C41—C42—C43	−175.56 (16)
C21—P1—C11—C16	133.67 (14)	C41—C42—C43—C44	−0.4 (3)
Ag1—P1—C11—C16	8.77 (16)	C42—C43—C44—C45	−0.5 (3)
C31—P1—C11—C12	65.03 (15)	C43—C44—C45—C46	0.3 (3)
C21—P1—C11—C12	−43.97 (15)	C44—C45—C46—C41	0.7 (3)
Ag1—P1—C11—C12	−168.87 (11)	C42—C41—C46—C45	−1.6 (3)
C16—C11—C12—C13	2.8 (2)	P2—C41—C46—C45	175.61 (16)
P1—C11—C12—C13	−179.50 (13)	C41—P2—C51—C56	−11.67 (17)
C11—C12—C13—C14	−1.5 (3)	C61—P2—C51—C56	98.60 (16)
C12—C13—C14—C15	−1.1 (3)	Ag1—P2—C51—C56	−134.76 (14)
C13—C14—C15—C16	2.4 (3)	C41—P2—C51—C52	170.39 (13)
C14—C15—C16—C11	−1.0 (3)	C61—P2—C51—C52	−79.34 (14)
C12—C11—C16—C15	−1.6 (2)	Ag1—P2—C51—C52	47.30 (14)
P1—C11—C16—C15	−179.23 (13)	C56—C51—C52—C53	−0.6 (3)
C11—P1—C21—C22	121.60 (15)	P2—C51—C52—C53	177.44 (14)
C31—P1—C21—C22	12.36 (16)	C51—C52—C53—C54	0.3 (3)
Ag1—P1—C21—C22	−110.96 (14)	C52—C53—C54—C55	0.4 (3)
C11—P1—C21—C26	−57.49 (14)	C53—C54—C55—C56	−0.8 (3)
C31—P1—C21—C26	−166.73 (13)	C54—C55—C56—C51	0.4 (3)
Ag1—P1—C21—C26	69.95 (14)	C52—C51—C56—C55	0.2 (3)
C26—C21—C22—C23	1.7 (3)	P2—C51—C56—C55	−177.65 (14)
P1—C21—C22—C23	−177.36 (13)	C41—P2—C61—C66	−106.80 (14)
C21—C22—C23—C24	0.7 (3)	C51—P2—C61—C66	145.35 (14)
C22—C23—C24—C25	−2.3 (3)	Ag1—P2—C61—C66	30.55 (16)
C23—C24—C25—C26	1.6 (3)	C41—P2—C61—C62	76.64 (16)
C24—C25—C26—C21	0.8 (3)	C51—P2—C61—C62	−31.20 (16)
C22—C21—C26—C25	−2.4 (3)	Ag1—P2—C61—C62	−146.00 (13)
P1—C21—C26—C25	176.72 (14)	C66—C61—C62—C63	−0.7 (3)
C11—P1—C31—C32	−9.27 (17)	P2—C61—C62—C63	175.88 (14)
C21—P1—C31—C32	95.60 (15)	C61—C62—C63—C64	0.2 (3)
Ag1—P1—C31—C32	−139.30 (13)	C62—C63—C64—C65	0.4 (3)
C11—P1—C31—C36	168.20 (13)	C63—C64—C65—C66	−0.7 (3)
C21—P1—C31—C36	−86.92 (14)	C64—C65—C66—C61	0.2 (3)
Ag1—P1—C31—C36	38.18 (14)	C62—C61—C66—C65	0.4 (3)
C36—C31—C32—C33	0.1 (3)	P2—C61—C66—C65	−176.21 (14)

### Hydrogen-bond geometry (Å, º)

Cg2 is the centroid of the C11–C16 ring.

**Table:**

*D*—H···*A*	*D*—H	H···*A*	*D*···*A*	*D*—H···*A*
N1—H1*A*···O3^i^	0.86	2.02	2.877 (2)	180
N1—H1*B*···O3^ii^	0.86	2.17	2.921 (2)	145
N2—H2···O1^i^	0.86	1.97	2.823 (2)	171
C35—H35···*Cg*2^iii^	0.93	2.97	3.746 (2)	142
C54—H54···*Cg*2^iv^	0.93	2.82	3.531 (2)	134

Symmetry codes: (i) *x*−1, *y*, *z*; (ii) −*x*+1, −*y*+1, −*z*+2; (iii) −*x*+1, *y*+1/2, −*z*+3/2; (iv) −*x*+1, −*y*+2, −*z*+2.
